# Editorial: Multifaceted cannabinoids: regulators of normal and pathological function in metabolic and endocrine organs, volume II

**DOI:** 10.3389/fendo.2024.1503017

**Published:** 2024-10-17

**Authors:** Rosaria Meccariello, Kanikkai Raja Aseer, Morvarid Kabir, Antonietta Santoro

**Affiliations:** ^1^ Department of Medical, Human Movement and Well-Being Sciences, University of Naples Parthenope, Naples, Italy; ^2^ Laboratory of Clinical Investigation, National Institute on Aging, National Institutes of Health, Baltimore, MD, United States; ^3^ Cedars Sinai Medical Center, Department of Medicine, Los Angeles, CA, United States; ^4^ Department of Medicine, Surgery and Dentistry, University of Salerno, Baronissi, Italy

**Keywords:** endocannabinoids, endocannabinoid system, phytocannabinoids, metabolic and endocrine tissues, gut aging, reproduction, energy balance

The endocannabinoid system (ECS) is one of the key modulators within the nervous system. It comprises the plant-derived phytocannabinoids, endogenous cannabinoids, endocannabinoid like substances, biosynthetic and hydrolyzing enzymes, classical and non-classical endocannabinoid receptors (1). Endocannabinoids were originally discovered in the brain as fundamental regulators of synaptic transmission. They are lipid mediators synthesized in different cell types. (2). By selectively activating different cannabinoid receptors, they exert a wide range of effects in the body, thus being capable to modulate cell proliferation, differentiation, and death, hormonal secretion, inflammation, energy metabolism, nociception also in peripheral tissues (3–8). Hence, there is growing interest in the ECS as a potential therapeutic target in metabolic, reproductive, neurological or cardiovascular disease treatment. In parallel, the recreational use of phytocannabinoids and their legalization for medical use in several countries, raise the question of how altering of this endogenous system may impact health and disease (9–11).

Nevertheless, the growing incidence of metabolic dysfunctions, overweight and obesity, infertility, and other related conditions in industrialized countries (12, 13), raises the question to the role of endocannabinoids in metabolic and endocrine tissues. In this regard, this Research Topic includes four manuscripts aimed at expanding our understanding of molecular mechanisms underlying the metabolic and endocrine action of the endocannabinoids.

The first review article included in this Research Topic by Bielawiec et al., is focused on the possible use of phytocannabinoids [i.e., 19-Tetrahydrocannabinol (19-THC), 19-Tetrahydrocannabivarin (19-THCV), Cannabinol (CBN), Cannabidivarin (CBDV), Cannabigerol (CBG), Cannabichromene (CBC), Cannabidiol (CBD)] in the treatment of insulin resistance and obesity. Particular attention has focused on CBD, the most widely distributed non-psychoactive compound in the cannabis plant, with promising therapeutic application in diseases and pain management (14). In pre-clinical studies, CBD exhibits well-established anti-inflammatory, antioxidant, anti-convulsant, anti-psychotic, and also potential anti-obesity properties. Thus, CBD is at the center of interest for its ability to increase intracellular lipolysis and mitochondrial activity in the liver and adipose tissue. CBD can inhibit liver steatosis, reduce inflammatory response and prevent weight gain. For this reason, it has been suggested as a potential treatment for preventing and reducing pancreatic damage associated with obesity and insulin resistance, as well as for preventing or treating diabetic complications.

The second manuscript by Lee et al. is a research article that investigates the possible involvement of CB1 signaling in gut aging. In a human and rat cellular model of senescent intestinal epithelial cells induced by hydrogen peroxide (H_2_O_2_) and hydroxyurea (HU), cellular permeability was evaluated by transepithelial electrical resistance (TEER) measurement along with the expression of CB1 and tight junction proteins. The expression of CB1 and zonula occludens-1 (ZO-1) was decreased in the small intestine of aged rats compared to that of young rats, with consequent reduced TEER values in senescent cells. *In silico* miRNA analysis and combined treatments with HU and CB1 agonist ACEA revealed the involvement of a molecular signaling pathway down-regulating CB1 and inducing the up regulation of *miR191-5P* that target ZO-1, and the activation of NF-kB p65 in gut senescence. Hence, the aging-induced reduction of CB1 leads to increased intestinal permeability and decreased ZO-1 expression via upregulation of *miR-191-5p* and NF-kB p65 activation.

The third research article by Marino et al. adds insights into the interplay between the kisspeptin system (KS) and the ECS along the hypothalamus-pituitary-testis axis of mammals. Both the KS and the ECS centrally modulate reproduction by acting on the hypothalamic gonadotropin Releasing Hormone (GnRH), but with opposite effects (15, 16). Nevertheless, the possible interplay between the two systems along the reproductive axis has been deeply investigated in non-mammalian vertebrates only, and data in mammals remain limited (17, 18). Using *in vivo* treatments with Kisspeptin-10 (Kp10), AEA and AEA ± SR141716A (Rimonabant, a CB1 antagonist) from the peripubertal period to sexual maturation, the effects within the hypothalamus and testis were investigated, alongside to puberty related miRs, morpho-functional evaluation of brain/testis and markers of spermatogenesis progression. For the first time in mammals, this manuscript reports the modulation of the KS in both the hypothalamus and testis by AEA *via* CB1 and reveals the KP-dependent modulation of CB1 and FAAH in the testis also suggesting a KP involvement in the progression of spermatogenesis.

The last manuscript of the Research Topic by Sotzen et al., characterizes the effects of the impaired cannabinoid signaling on body weight gain and glucose metabolism in female mice, thus addressing a knowledge gap regarding the possible sex dimorphic activity of cannabinoid receptors in the modulation of energy balance. In this study, CB1^-/-^, CB2^-/-^, or CB1^-/-^/CB2^-/-^ knockout and wild-type mice were fed with a low (10% of calories from fat) or high-fat diet (45% of calories from fat) for six weeks. In female mice, deletion of CB1 alone had attenuated effects on body weight gain and thermogenesis, whereas deletion of CB2 or both CB1 and CB2 exhibited effects that were broadly consistent with reported findings on male mice.

Taken together, although limited to 4 manuscripts, this Research Topic of Frontiers in Endocrinology provides snapshots regarding the most relevant questions of cannabinoids within the metabolic/endocrine system with consequences on obesity and infertility opening new questions about cannabinoids function and mechanisms in body homeostasis. The Research Topic and herein papers provide also inputs on the potential of phytocannabiboids, particularly CBD, as therapeutic drugs in pancreatic damage related to obesity and insulin resistance, and insights on the involvement of the ECS in the modulation of gut aging, reproductive axis, and its sex dimorphic activity in energy balance, highlighting the potential pharmacological applications of ECS modulation.
